# Basic and clinical immunology – 3015. Nonspecific defense factors in premature infants with pneumonia

**DOI:** 10.1186/1939-4551-6-S1-P191

**Published:** 2013-04-23

**Authors:** Kamola Alimova, Nigora Fayzullaeva, Furkat Muhitdinovich Shamsiev

**Affiliations:** 1 Republican Scientific and Practical Medical Center of Pediatrics Uzbekistan, Tashkent, Uzbekistan; 2 Institute of Immunology, Tashkent, Uzbekistan

## 

Neonatal pneumonia diagnosed in 0.5 - 1.0% of full-term and 10 - 15% of premature babies. Of neonatal pneumonia contributes to failure resulting from maternal immunity factors, which affects the function of the immune system and, in particular, the mechanisms of innate immunity.

Lactoferrin - one of the factors of innate immunity directed against a range of bacteria, viruses and fungi. The aim of the study was to examine the levels of lactoferrin in term and preterm infants with pneumonia. The observation 84 infants: 55 - neonatal pneumonia patients and 29 healthy. In the patients with pneumonia were 36 full-term and 19 preterm infants. The concentration of lactoferrin in the serum were determined by enzyme immunoassay system "Lactoferrin Strip" ("Vector-Best", Novosibirsk). Statistical processing of the results was performed using the application.

Clinical examination showed that the most frequently encountered one-and two-sided polysegmentary pneumonia, at least - pneumopathy and unilateral focal pneumonia. Pneumopathy, characterized by respiratory failure and oxygen dependence, often associated with CNS (in 62% of cases with cerebral ischemia II degree, and 30% - with intra-ventricular hemorrhage I-II level) in 43% of unilateral and 55% - bilateral pneumonia developed against the background pneumopathy. The clinical course of neonatal pneumonia, herpes virus, in combination infektsiny, was the most difficult. Bilateral focal pneumonia occurred in 7.1% (p ƒ¬ 0,01) cases sided polysegmental - in 42.9% (p ƒ¬ 0,01), polysegmental bilateral pneumonia - by 16.9% (p ƒ¬ 0,01) cases. In the blood of newborns with neonatal pneumonia lactoferrin level was 100 times lower than that of a healthy adult. When herpes infection lactoferrin concentration was higher (p ƒ¬ 0,01) at the beginning and at the height of the disease, but the convalescence against the normalization of clinical data in patients with pneumonia, herpes virus, it remained significantly higher than normal. Increasing lactoferrin concentration in the serum at neonatal pneumonia caused, apparently, not vacuumed from the intestine, and the collapse of abundant neutrophil granulocytes. Ie increasing the concentration of lactoferrin - is a sign of pathogenic response of myeloid blood to infection of the lungs.

